# Timing, Composition, and Clinical Correlates of Immunotherapy Response in GAD65 Antibody-Associated Epilepsy: A Literature-Derived Patient-Level Analysis of 375 Published Cases

**DOI:** 10.3390/neurolint18060121

**Published:** 2026-06-22

**Authors:** József Janszky, József Janszky, Réka Horváth

**Affiliations:** 1 Department of Psychiatry and Psychotherapy, Medical School, University of Pécs, Rét utca 2, H-7623 Pécs, Hungary; janszky.jozsef2@pte.hu; 2 Department of Neurology, Medical School, University of Pécs, Rét utca 2, H-7623 Pécs, Hungary; janszky.jozsef@pte.hu; 3 HUN-REN-PTE Clinical Neuroscience MR Research Group, Rét utca 2, H-7623 Pécs, Hungary

**Keywords:** GAD65 antibody, autoimmune epilepsy, temporal lobe epilepsy, immunotherapy, rituximab, seizure outcome, drug resistance

## Abstract

Objective: Glutamic acid decarboxylase 65 (GAD65) antibody-associated epilepsy often presents as chronic focal epilepsy, usually with temporal lobe predominance, marked drug resistance, and inconsistent response to first-line immunotherapy. We assembled a large, harmonized, and literature-derived patient-level cohort to examine whether immunotherapy timing and regimen composition were associated with seizure outcome and to identify clinically meaningful prognostic signals. Methods: We performed a literature-derived patient-level analysis of 375 unique published cases linked to 132 contributory source publications from an audited full-text register of 166 reviewed studies. Descriptive analyses used the whole cohort. Treatment-response analyses assessed seizure outcome at the first evaluable post-immunotherapy assessment and at the last follow-up. Good seizure outcome was defined as seizure freedom and/or ≥50% seizure reduction. The primary timing comparison contrasted early treatment, defined as immunotherapy within 6 months of symptom onset, with late treatment, defined as immunotherapy after more than 12 months; four cases treated in the intermediate >6 to ≤12 month window were retained for descriptive timing summaries but excluded from the primary comparison. Statistical testing used the Fisher exact, Chi-square, Mann–Whitney U, and prespecified clustered logistic sensitivity analyses where appropriate. Results: The pooled phenotype was predominantly female, usually temporal-lobe-based, and frequently drug-resistant, with common autoimmune comorbidity and heterogeneous MRI abnormalities. Among timing-evaluable treated cases, earlier immunotherapy showed a class-specific, exploratory signal rather than a uniform regimen-independent effect. In rituximab/CD20-directed regimens, early treatment was associated with a higher rate of good seizure outcome than late treatment at both the first post-immunotherapy assessment and last follow-up (93.8% vs. 50.0%; risk difference [RD]: 43.8 percentage points; 95% CI: 7.7 to 72.7). A similar pattern was observed in the broader escalation group (94.4% vs. 55.6%; RD: 38.9 percentage points; 95% CI: 6.3 to 68.1). By contrast, steroid-containing regimens showed no clear early-versus-late advantage (84.6% vs. 88.2%; RD: −3.6 percentage points; 95% CI: −18.4 to 20.1). Shorter epilepsy duration before immunotherapy and absence of established drug resistance were the most clinically meaningful favorable baseline features. Significance: In GAD65 antibody-associated epilepsy, the therapeutic window may be most relevant for escalation strategies rather than for steroid-containing first-line regimens. However, these class-specific findings are exploratory and hypothesis-generating. They derive from non-randomized, literature-derived data and may reflect treatment intensity, center practice, publication era, and confounding by indication rather than isolated regimen superiority. Prospective collaborative registries with standardized longitudinal seizure outcome measures are needed to validate these observations.

## 1. Introduction

Glutamic acid decarboxylase 65 (GAD65) antibody-associated epilepsy occupies a distinctive position within autoimmune epilepsies. Unlike many neuronal surface antibody syndromes, it commonly evolves into chronic focal epilepsy, usually with temporal lobe predominance, high seizure burden, frequent drug resistance, and variable cognitive or psychiatric involvement. High serum titers and intrathecal synthesis increase diagnostic confidence, but the clinical syndrome remains heterogeneous, ranging from isolated epilepsy presentations to limbic encephalitic phenotypes, status epilepticus, and overlap with other GAD65-associated neurologic autoimmunities [1,2,3,4,5,6,7].

Glutamic acid decarboxylase 65 is one of the two major isoforms of glutamic acid decarboxylase and contributes to gamma-aminobutyric acid (GABA) synthesis in presynaptic terminals. In epilepsy, GAD65 antibodies are generally interpreted as markers of a broader immune process rather than as evidence of a simple directly pathogenic antibody mechanism. High antibody titers and CSF or intrathecal GAD65 positivity increase diagnostic specificity. The same autoimmune background can coexist with systemic autoimmune diseases, particularly type 1 diabetes mellitus and autoimmune thyroid disease. For this reason, epilepsy presentations associated with GAD65 antibodies often require integrated neurologic and systemic autoimmune assessment [1,5,7].

From a therapeutic standpoint, GAD65 antibody-associated epilepsy is challenging. Corticosteroids, intravenous immunoglobulin, and plasma exchange may benefit some patients, but responses are often incomplete, transient, or absent, and treatment response is less predictable than in typical neuronal surface antibody encephalitides [2,3,4,5,6,8,9,10,11]. This therapeutic uncertainty likely reflects both biology and timing. GAD65 is an intracellular antigen, neuropathologic studies suggest prominent T-cell-rich limbic inflammation rather than a purely antibody-effector process, and many patients start immunotherapy only after months or years of established epileptogenesis [7,9,10,11].

Most available evidence still comes from single cases, small series, or syndrome-overlap cohorts. This literature is clinically rich but analytically fragmented: treatment sequences are described inconsistently, treatment timing is often imprecise, and long-term seizure outcomes are rarely reported in a comparable way across studies. As a result, three clinically important questions remain unresolved. First, does earlier immunotherapy improve seizure outcome in GAD65 antibody-associated epilepsy? Second, does timing matter equally across first-line and escalation regimens? Third, which baseline clinical or paraclinical features are most useful when counseling patients about prognosis?

To address these questions, we assembled a harmonized patient-level database of published GAD65 antibody-associated epilepsy cases, audited the contributory full-text source register, and analyzed the cohort at both descriptive and outcome-focused levels. Our aims were to characterize the pooled phenotype, examine immunotherapy composition and timing in relation to short- and long-term seizure outcomes, evaluate epilepsy duration as a potential therapeutic window marker, and identify clinically meaningful correlates of favorable outcome. We also explored whether sodium-channel-blocking antiseizure medication exposure modified the observed association between immunotherapy timing and seizure outcome.

## 2. Methods

### 2.1. Study Design, Literature Search, and Case Identification

We performed a literature-derived patient-level analysis of published cases of GAD65 antibody-associated epilepsy. The literature search was conducted between 1 May 2025 and 1 April 2026, with the final search update performed on 1 April 2026, before initial manuscript submission on 23 April 2026. PubMed/MEDLINE, Embase, Scopus, and Google Scholar were searched, and reference lists of eligible articles and relevant reviews were screened manually for additional records. Search terms combined GAD65 autoimmunity and epilepsy-related phenotypes: (“glutamic acid decarboxylase” OR “glutamic acid decarboxylase 65” OR GAD65 OR anti-GAD OR anti-GAD65) AND (epilep* OR seizure* OR “temporal lobe epilepsy” OR “limbic encephalitis” OR “autoimmune encephalitis” OR “status epilepticus” OR musicogenic).

Original reports were eligible if they described individual GAD65-positive patients with epilepsy or recurrent seizures and provided sufficiently granular patient-level clinical data extractable from the main text, tables, or Supplementary Material. Review articles without de novo cases, aggregate-only cohorts without individual patient data, duplicate or overlapping publications without uniquely attributable cases, and reports falling outside the final epilepsy-focused case definition were excluded. Cases coded as generalized epilepsy, idiopathic generalized epilepsy, or juvenile myoclonic epilepsy were manually rechecked against the source publication and retained only when both true GAD65 positivity and attributable epilepsy data could be confirmed.

**Figure 1:**
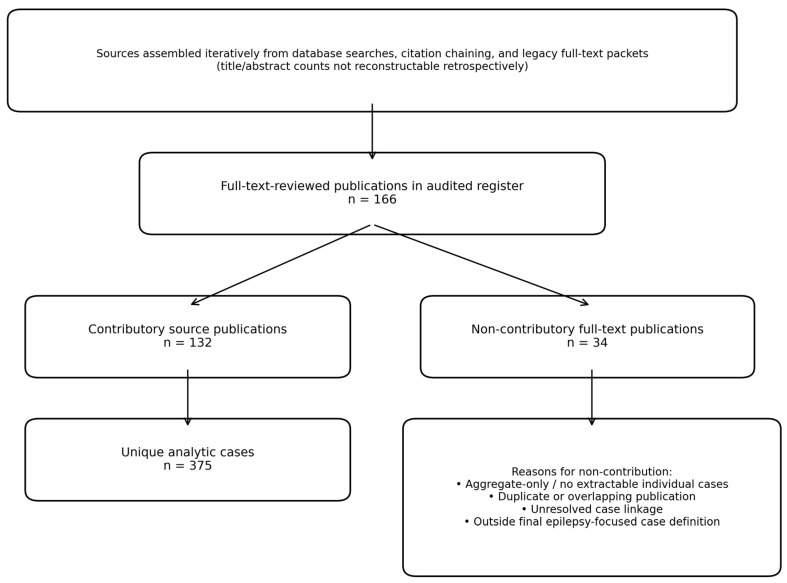
PRISMA-informed retrospective audit flow of full-text-reviewed publications contributing to the literature-derived patient-level GAD65 antibody-associated epilepsy database.

Because the source database was assembled iteratively through database searches, citation chaining, and legacy full-text source packets, a conventional retrospective title and abstract screening cascade could not be reconstructed with sufficient confidence. We therefore report a PRISMA-informed retrospective audit flow of full-text-reviewed publications (Figure 1), rather than a post hoc full screening diagram.

The working study register contained 166 full-text-reviewed publications. A source publication was considered contributory only if at least one unique patient-level case meeting the final case definition could be linked with confidence. Potential duplicate or overlapping cases were adjudicated through a prespecified multistep process. Candidate overlaps were first flagged on the basis of shared author groups, center or country, recruitment period, or unusually similar phenotype descriptions. Flagged records were then compared across age at onset or presentation; sex, seizure semiology; epilepsy laterality; MRI, EEG, PET, and CSF findings; GAD65 titer or intrathecal status; coexisting autoimmunity; immunotherapy chronology; surgery or neuromodulation history; and follow-up narrative.

When overlap was considered likely, the most information-rich report was retained as the anchor record. Nonredundant details from linked reports were incorporated only when they could be attributed with confidence to the same patient without double counting. Publications with aggregate-only data, unresolved linkage, or no unique cases after adjudication were retained in the audited full-text register but excluded from the analytic case set.

After case-level verification and overlap adjudication, 132 publications contributed 375 unique cases. The remaining 34 full-text-reviewed publications did not contribute unique analytic cases because they were aggregate-only, duplicate or overlapping; lacked secure case-level linkage; or fell outside the final epilepsy-focused case definition. Included source publications are listed in Supplementary File S2.

### 2.2. Data Extraction and Analysis Groups

Data were abstracted case by case into a structured workbook that included study metadata; demographics; age at onset; epilepsy phenotype; seizure burden and status epilepticus; cognitive, psychiatric, and other neurologic manifestations; EEG, MRI, PET, CSF and serologic findings; autoimmune comorbidities; antiseizure medications; immunotherapy sequence and timing; surgery or neuromodulation; follow-up duration; and seizure, cognitive, and radiologic outcomes. When a field was not explicitly reported or could not be extracted with confidence, it was coded as missing rather than assumed absent.

The final database contained 375 unique cases from 132 contributory source publications published between 1988 and 2026. The audited full-text register also retained 34 reviewed publications that did not contribute unique analytic cases. The analyses were performed in two predefined analysis groups. The full cohort was used for descriptive phenotype analyses, broad treatment summaries, timing overviews, and exploratory surgery and neuromodulation summaries. A durable-outcome subset of 95 cases from 53 publications was used for analyses requiring comparable longitudinal seizure outcome reporting and a nonmissing binary durable-outcome classification.

### 2.3. Definitions and Outcomes

Adult-onset disease was defined as seizure onset at ≥18 years, and pediatric-onset disease as seizure onset before 18 years. Drug resistance was defined according to the 2010 ILAE criteria [12]. Very high GAD65 antibody titer in serum and/or CSF, intrathecal GAD65 positivity, definite temporal lobe epilepsy, multifocality, bilateral temporal MRI involvement, inflammatory MRI abnormalities, unilateral hippocampal sclerosis, and MRI-negative status were assigned from structured case-level variables when available. Extra-temporal MRI involvement was coded conservatively and only when explicit non-temporal neuroanatomic structures were described.

Immunotherapy exposure was defined as at least one documented immunotherapy treatment. For timing analyses, treatment timing was measured from symptom onset to the first recorded immunotherapy. Timing was summarized descriptively in three groups: ≤6 months, >6 to ≤12 months, and >12 months. The prespecified primary comparison contrasted early treatment, defined as immunotherapy within 6 months of symptom onset, with late treatment, defined as immunotherapy after more than 12 months. Cases treated in the intermediate >6 to ≤12 month window were included in descriptive timing summaries but excluded from the primary early-versus-late comparison.

The treatment analyses were structured in two complementary ways. First, for descriptive initiating regimen summaries, each treated case was assigned to a single mutually exclusive category when the initial regimen could be identified from the structured fields. These categories were steroid plus intravenous immunoglobulin, steroid only, intravenous immunoglobulin only, rituximab-based therapy, maintenance antimetabolite-based therapy, steroid plus plasma exchange or immunoadsorption, and apheresis-based therapy. Second, for component-class timing analyses, categories were intentionally allowed to overlap. In these analyses, a single case could contribute to more than one treatment-component row if the regimen included multiple immunotherapy components. Therefore, component-class denominators and row totals should not be interpreted as additive patient groups.

The primary efficacy endpoint was good seizure outcome, defined as seizure freedom and/or at least 50% seizure reduction. Outcomes were assessed at two clinically relevant time points: the first evaluable post-immunotherapy seizure assessment and the last available follow-up. Second-line or other immunomodulatory exposure included rituximab, azathioprine, mycophenolate mofetil, cyclophosphamide, tacrolimus, tocilizumab, and basiliximab. To explore the potential influence of background antiseizure medication, sodium-channel-blocking exposure was defined as treatment with lamotrigine, carbamazepine, oxcarbazepine, or valproate at or around immunotherapy initiation.

### 2.4. Statistics

Analyses used complete-case denominators. Binary comparisons were assessed using the two-sided Fisher exact or Chi-square tests, as appropriate, and skewed continuous variables were compared using Mann–Whitney U tests. For the principal binary comparisons, unadjusted absolute risk differences with 95% confidence intervals were added to *p* values to show both effect size and precision. These differences were calculated as early minus late, or exposed minus comparator, depending on the analysis.

Clustered logistic regression with study-level robust standard errors was used as a sensitivity analysis in the durable-outcome subset. Non-age predictors were prespecified to be adjusted for adult onset and drug resistance, whereas adult onset was adjusted for drug resistance alone. Benjamini–Hochberg false-discovery-rate correction was applied to the exploratory baseline predictor screen. Because this was a literature-derived observational synthesis with variable reporting completeness, all treatment comparisons were interpreted as associative rather than causal.

## 3. Results

### 3.1. Cohort Phenotype

The audited study register contained 166 full-text-reviewed publications, of which 132 contributed at least one unique case after overlap adjudication and 34 were noncontributory after full-text review (Figure 1). The final database comprised 375 unique cases from 132 source publications published between 1988 and 2026. Sex was reported in 347 cases, of whom 253/347 (72.9%) were female. Age at onset was available in 249 cases, with a mean onset age of 25.0 years (range 1.0–80.0). Adult-onset disease was documented in 170 cases and pediatric onset in 91. Among adult-onset cases with known sex, 134/169 (79.3%) were female; among pediatric-onset cases, 68/91 (74.7%) were female (Table 1).

Drug-resistance status was informative in 210 cases, and 179/210 (85.2%) met the criteria for drug-resistant epilepsy. Autoimmune thyroid disease only was recorded in 25 cases, type 1 diabetes mellitus only in 16, and both conditions in 23; paired comorbidity reporting was incomplete, but the overall pattern supported frequent systemic autoimmunity (Table 2B). Definite temporal lobe epilepsy was identified in 223/375 cases, unilateral temporal lobe epilepsy in 99/375, bilateral temporal MRI involvement in 63/375, extra-temporal MRI involvement in 44/375, inflammatory MRI abnormalities in 135/375, definite unilateral hippocampal sclerosis in 46/375, MRI-negative status in 99/375, and multifocal epilepsy or multifocal involvement in 65/375 (Table 2A).

A very high GAD65 antibody titer was documented in 224/375 cases and intrathecal GAD65 positivity in 87/375. Additional non-GAD antibody positivity was reported in 75/375 cases, most commonly anti-thyroid peroxidase, anti-thyroglobulin, and AMPAR antibodies. Tumor screening was documented in 114/375 cases and was positive in 18/114. A history of status epilepticus was reported in 50/375 cases (Table 2A).

### 3.2. Immunotherapy Exposure and Timing Overview

**Figure 2:**
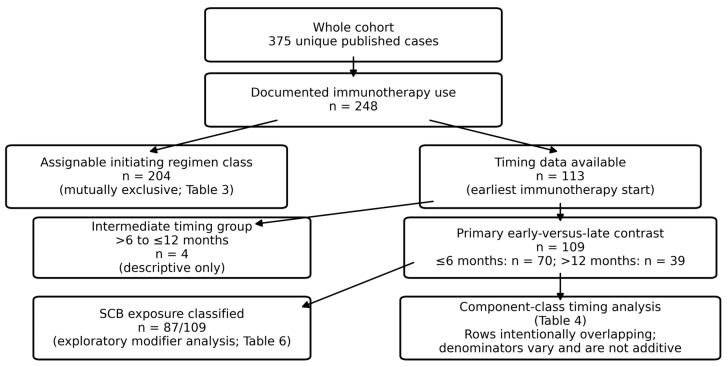
Analytic flow from treated cases to regimen composition and timing analyses. The figure summarizes how the treated cohort was narrowed for the different immunotherapy analyses. Of the 248 treated patients, 204 had an identifiable initial immunotherapy regimen, and 113 had sufficient timing information. Because component-based treatment categories were not mutually exclusive, a patient could contribute to more than one regimen component analysis if the regimen included multiple immunotherapy components. This explains why denominators differ across the regimen composition and timing analyses.

Immunotherapy was documented in 248/375 cases. The initial immunotherapy regimen could be classified in 204 of 248 treated patients (82.3%): steroid plus intravenous immunoglobulin in 104, steroid only in 37, intravenous immunoglobulin only in 22, rituximab-based therapy in 16, maintenance antimetabolite-based therapy in 15, steroid plus plasma exchange/immunoadsorption in 7, and apheresis-based therapy in 3 (Table 3). Timing from symptom onset was available in 113/248 treated cases; 70/113 (61.9%) started within 6 months, 4/113 (3.5%) in the intermediate >6 to ≤12 month window, and 39/113 (34.5%) after 12 months. The intermediate timing group was retained for descriptive completeness but excluded from the prespecified early-versus-late contrast. The analytic flow from treated cases to regimen composition and timing analyses is summarized in Figure 2.

### 3.3. Primary Timing Analyses Using the Prespecified Early Window

**Figure 3:**
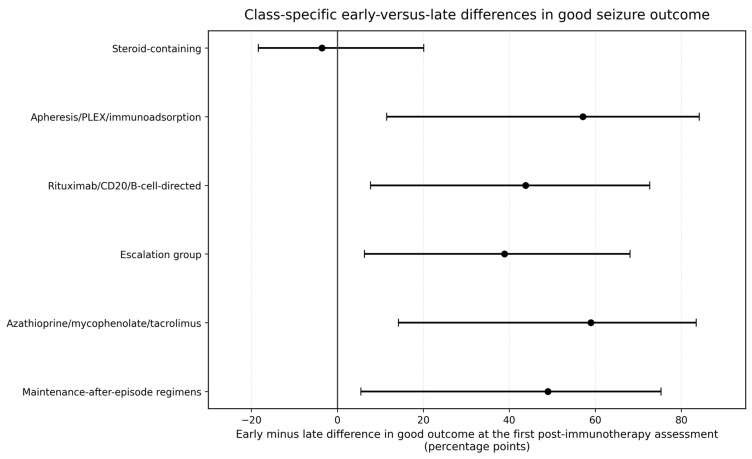
Class-specific absolute differences in good seizure outcome at the first evaluable post-immunotherapy assessment for early-versus-late immunotherapy. Points show unadjusted absolute differences, and horizontal lines show 95% confidence intervals. Positive values favor earlier treatment. Estimates are exploratory and based on within-class comparisons.

For the prespecified early-versus-late comparison, 109 treated cases had sufficient timing information: 70 patients received immunotherapy within 6 months of symptom onset, and 39 received immunotherapy after more than 12 months. The four patients treated in the intermediate >6 to ≤12 month window were retained in the descriptive timing summary but excluded from this primary comparison. Overall, earlier treatment showed a numerical advantage, particularly at the first post-immunotherapy assessment. However, this pattern was not consistent across all treatment regimens, so we next examined early-versus-late outcomes within each immunotherapy component class (Table 4), with the corresponding class-specific absolute differences shown in Figure 3.

### 3.4. Class-Specific Immunotherapy Timing and Outcome

Because treatment-component classes intentionally overlap, Table 4 reports within-class early-versus-late contrasts rather than mutually exclusive patient strata and now presents effect estimates with 95% confidence intervals in addition to *p* values. Among steroid-containing regimens, early treatment was not associated with better seizure outcome than late treatment either at the first post-immunotherapy assessment (44/52 [84.6%] vs. 15/17 [88.2%]; RD: −3.6 percentage points; 95% CI: −18.4 to 20.1; *p* = 1.000) or at last follow-up (44/53 [83.0%] vs. 15/17 [88.2%]; RD: −5.2 percentage points; 95% CI: −20.1 to 18.7; *p* = 1.000). By contrast, early apheresis/immunoadsorption-containing regimens showed a favorable early signal at the first post-immunotherapy assessment (8/8 [100%] vs. 3/7 [42.9%]; RD: 57.1 percentage points; 95% CI: 11.5 to 84.2; *p* = 0.0256), although this pattern did not clearly persist at last follow-up.

The clearest exploratory timing signal emerged in rituximab/CD20-directed regimens. Good early outcome occurred in 15/16 (93.8%) early-treated versus 4/8 (50.0%) late-treated cases (RD: 43.8 percentage points; 95% CI: 7.7 to 72.7; *p* = 0.0277), and the same proportions were observed at last follow-up (RD: 43.8 percentage points; 95% CI: 7.7 to 72.7; *p* = 0.0277). A similar pattern was present in the broader escalation group defined by cyclophosphamide and/or rituximab/CD20-directed therapy and/or tocilizumab: 17/18 (94.4%) versus 5/9 (55.6%), respectively, at both early and late outcome time points (RD: 38.9 percentage points; 95% CI: 6.3 to 68.1; *p* = 0.0297 at both time points).

Among azathioprine/mycophenolate/tacrolimus-containing regimens, early treatment was also associated with a more favorable early seizure outcome (12/13 [92.3%] vs. 2/6 [33.3%]; RD: 59.0 percentage points; 95% CI: 14.2 to 83.5; *p* = 0.0173), whereas the last-follow-up comparison remained less precise. Maintenance-after-episode regimens showed the same directional pattern. Given the small late-treatment cell counts in several escalation strata, these class-specific associations should be interpreted as exploratory and hypothesis-generating rather than as proof of regimen superiority. Within the early-treatment subgroup, broader regimen intensity was associated with better early seizure outcomes: the median number of immunotherapy agents was three in cases with good early outcomes versus two in those without good early outcomes (*p* = 0.0201). This effect weakened at last follow-up and did not remain robust.

### 3.5. Epilepsy Duration and Clinico-Paraclinical Correlates of Outcome

Shorter epilepsy duration before immunotherapy was associated with better seizure outcome at both evaluated time points. At the first post-immunotherapy assessment, patients with a good seizure outcome had a shorter median pre-treatment epilepsy duration than those without a good outcome (1.33 years [IQR 0.17–5.0] vs. 5.0 years [IQR 1.0–14.25]; *p* = 0.00086). The same direction was observed at the last follow-up, although the association was weaker (1.5 years [IQR 0.2–8.0] vs. 4.0 years [IQR 1.0–9.0]; *p* = 0.0241) (Table 5).

In the exploratory baseline feature screen, drug resistance was the most clinically coherent unfavorable feature. At the first post-immunotherapy assessment, good seizure outcome was less frequent in drug-resistant cases than in non-drug-resistant cases (54/82 [65.9%] vs. 18/18 [100.0%]; absolute difference: −34.1 percentage points; 95% CI: −44.9 to −14.2; *p* = 0.0026). A similar pattern was present at the last follow-up (65/89 [73.0%] vs. 19/19 [100.0%]; absolute difference: −27.0 percentage points; 95% CI: −37.0 to −8.3; *p* = 0.0063). After false-discovery-rate correction, no screened baseline feature met the conventional q < 0.05 threshold; however, drug resistance remained the most consistent near-threshold clinical signal at the early outcome assessment (q = 0.056).

Documented tumor screening was nominally associated with good outcomes at both time points, but this finding was difficult to interpret biologically and most likely reflected reporting or clinical-workup differences rather than a stable prognostic marker. History of status epilepticus and selected MRI features showed only isolated nominal associations and were not retained as consistent predictors after multiplicity assessment.

### 3.6. Sodium-Channel-Blocking Antiseizure Medication Exposure

Among the 109 treated cases included in the early-versus-late timing analysis, sodium-channel-blocking (SCB) antiseizure medication status could be classified in 87 cases: 42 were SCB-exposed and 45 were not exposed. Outcome-evaluable denominators were smaller and varied by timing subgroup and outcome time point. SCB exposure was not associated with a significant difference in seizure outcome either among early-treated cases or among late-treated cases. Interaction models also did not support a significant modifying effect of SCB exposure on the association between immunotherapy timing and seizure outcome (interaction *p* = 0.788 for the first post-immunotherapy assessment and *p* = 0.314 for last follow-up) (Table 6).

### 3.7. Surgery and Neuromodulation

Surgery or neuromodulation was documented in 55 patients, corresponding to 56 procedures. The recorded interventions were mainly amygdalohippocampal or other temporal resection-type procedures (43/56) and vagus nerve stimulation (VNS) implantations (11/56), with isolated cases of responsive neurostimulation (RNS) and laser interstitial thermal therapy (LITT). Outcome reporting was limited: an explicit benefit category was available for only 19 procedures. In these reported cases, durable seizure freedom was uncommon, whereas partial benefit was more frequently described than complete remission.

Pathology was informative in 14 resections. Active inflammation was reported in nine cases and hippocampal sclerosis in six; these findings were not mutually exclusive, indicating that immune activity and chronic mesial temporal structural injury may coexist in operated patients with GAD65 antibody-associated epilepsy. Because of the limited and inconsistently reported outcome and pathology data, no formal comparative efficacy analysis of surgery or neuromodulation was attempted. We therefore present these findings as a descriptive adjunct to the central therapeutic window question. The coexistence of active inflammation and chronic structural injury may help to explain why both immunotherapy response and surgical outcomes are variable in this syndrome. Aggregate details are provided in Supplementary File S1.

## 4. Discussion

This literature-derived patient-level synthesis supports the view that GAD65 antibody-associated epilepsy is a clinically recognizable but therapeutically challenging syndrome. Across 375 published cases from 132 contributory source publications, the pooled phenotype was predominantly female, usually temporal-lobe-based, commonly drug-resistant, and frequently associated with systemic autoimmunity. The central treatment-related finding was not a universal “earlier is always better” effect, but a class-specific, hypothesis-generating signal: earlier treatment appeared most favorable in rituximab/CD20-directed and broader escalation regimens, whereas steroid-containing regimens showed no clear early-versus-late advantage.

This signal should not be interpreted as evidence that rituximab is universally superior, or that GAD65 antibody-associated epilepsy is primarily a B-cell-mediated disorder. Neuropathologic and clinical studies instead support a more complex biology, including T-cell-rich limbic inflammation, intrathecal immune activation, and chronic hippocampal injury. The rituximab/escalation findings are therefore best interpreted as suggesting that treatment timing and treatment intensity may interact in selected patients. However, because these data are non-randomized and literature-derived, the observed associations remain vulnerable to center practice, era effects, treatment selection, and confounding by indication.

The strong association between shorter epilepsy duration before immunotherapy and better early seizure outcome reinforces the concept of a therapeutic window. Clinically, treatment delay may matter not only because seizures continue, but also because the disease may evolve from a potentially modifiable immune-active epilepsy into a more fixed mesial temporal epilepsy phenotype. Our data also suggest that absence of established drug resistance is the most clinically coherent favorable baseline feature.

The descriptive phenotype data are clinically instructive. Although the syndrome was strongly weighted toward temporal lobe epilepsy, bilateral temporal, multifocal, and MRI-negative presentations were not rare, and autoimmune comorbidity was common. These features support considering GAD65 antibody testing in selected patients with otherwise unexplained temporal or bitemporal epilepsy, particularly when accompanied by type 1 diabetes, autoimmune thyroid disease, cognitive symptoms, psychiatric change, musicogenic or other reflex features, or atypical MRI evolution [4,5,6,8,9,10]. The surgery and neuromodulation findings further support this framework. Selected patients may benefit from surgical or neuromodulatory interventions, but long-term seizure freedom appears uncommon, and available pathology data indicate that active inflammation and hippocampal sclerosis can coexist in operated patients [7,10,13]. This coexistence suggests that immune activity and fixed mesial temporal structural injury should not be viewed as mutually exclusive explanations for drug-resistant temporal lobe epilepsy in the GAD65 antibody-associated context.

The sodium-channel-blocking antiseizure medication analysis yielded a negative but clinically useful result. Although sodium-channel-blocking antiseizure medications are commonly used in focal autoimmune epilepsies and may have theoretical advantages in some inflammatory contexts, we found no robust evidence that they modified the association between immunotherapy timing and seizure outcome in GAD65 antibody-associated epilepsy. This finding does not argue against their symptomatic use. Rather, it suggests that the key therapeutic question in this syndrome may be less about the specific antiseizure medication backbone and more about timely recognition, appropriate immunotherapy selection, and when to escalate treatment.

## 5. Limitations

This study has several important limitations. First, it is a pooled analysis of published cases rather than a prospective registry, and is therefore vulnerable to publication bias, selection bias, variable follow-up, and confounding by indication. Reporting completeness differed substantially across key variables, including immunotherapy timing, seizure outcome, antibody testing results, and concomitant antiseizure medication exposure. The source database was assembled iteratively through database searches, citation chaining, and legacy full-text source packets. Therefore, a conventional retrospective title and abstract PRISMA flow could not be reconstructed with sufficient confidence; instead, we report an audited full-text register and a PRISMA-informed retrospective audit flow.

Several class-specific treatment analyses relied on small cell counts, particularly in late-treated escalation strata. These findings should therefore be regarded as exploratory and hypothesis-generating. Treatment-component classes were not mutually exclusive, because a single regimen could include more than one immunotherapy component. In addition, treatment allocation was nonrandom and likely influenced by center practice, publication era, disease severity, and clinician preference. Accordingly, the observed associations should inform clinical reasoning but should not be interpreted as causal evidence of regimen superiority.

The surgery and neuromodulation summary has additional limitations. Explicit outcome categories were available for only a minority of procedures, and pathology reporting was incomplete. This section should therefore be read as descriptive clinical context rather than as a formal efficacy analysis.

## 6. Conclusions

In this large literature-derived patient-level analysis, the central treatment question in GAD65 antibody-associated epilepsy is not simply whether immunotherapy is beneficial, but which patients may still have a modifiable autoimmune component and when escalation beyond first-line treatment should be considered. Earlier rituximab/CD20-directed and broader escalation strategies showed the most consistent favorable timing signals, whereas steroid-containing regimens did not show a comparable early-versus-late pattern. However, these class-specific findings are exploratory and hypothesis-generating, and should not be interpreted as evidence of regimen superiority.

Shorter epilepsy duration before immunotherapy and absence of established drug resistance were the most clinically meaningful favorable features. Together, these findings support earlier recognition of GAD65 antibody-associated epilepsy, careful consideration of timely escalation in selected patients, transparent reporting of immunotherapy timing and regimen composition, and prospective validation in collaborative registries using standardized longitudinal seizure outcome measures.

## Figures and Tables

**Table 1:** Cohort provenance and demographic characteristics.

Characteristics	Value (%)
Unique cases	375
Contributory source publications represented	132
Audited full-text-reviewed publications in source register	166
Full-text-reviewed but noncontributory after adjudication	34
Source publication year range	1988–2026
Sex data available	347/375 (92.5%)
Female sex	253/347 (72.9%)
Male sex	94/347 (27.1%)
Adult-onset cases (≥18 years)	170/375 (45.3%)
Pediatric-onset cases (<18 years)	91/375 (24.3%)
Female sex among adult-onset cases	134/169 (79.3%)
Male sex among adult-onset cases	35/169 (20.7%)
Female sex among pediatric-onset cases	68/91 (74.7%)
Male sex among pediatric-onset cases	23/91 (25.3%)
Age at disease onset, years, mean (range)	25.0 (1.0–80.0)

**Table 2:** (**A**) Clinical, MRI, serologic, and oncologic characteristics. (**B**) Cross-tabulation of autoimmune thyroid disease and type 1 diabetes mellitus status.

(**A**)					
**Characteristics**				**Value (%)**	
Drug-resistance information available				210/375 (56.0%)	
Drug-resistant epilepsy among informative cases				179/210 (85.2%)	
Autoimmune thyroid disease only				25/375 (6.7%)	
Type 1 diabetes mellitus only				16/375 (4.3%)	
Both autoimmune thyroid disease and type 1 diabetes mellitus				23/375 (6.1%)	
Definite temporal lobe epilepsy				223/375 (59.5%)	
Unilateral temporal lobe epilepsy				99/375 (26.4%)	
Bilateral temporal MRI involvement				63/375 (16.8%)	
Extra-temporal MRI involvement				44/375 (11.7%)	
Inflammatory MRI abnormalities				135/375 (36.0%)	
Definite unilateral hippocampal sclerosis				46/375 (12.3%)	
MRI-negative				99/375 (26.4%)	
MRI data unavailable				62/375 (16.5%)	
Multifocal epilepsy/multifocal involvement				65/375 (17.3%)	
Very high GAD65 titer (serum and/or CSF)				224/375 (59.7%)	
Intrathecal GAD65 positivity				87/375 (23.2%)	
Any additional structured antibody positivity				75/375 (20.0%)	
Most frequent additional structured antibody (anti-TPO)				34/375 (9.1%)	
Second most frequent additional structured antibody (anti-thyroglobulin)				17/375 (4.5%)	
Third most frequent additional structured antibody (AMPAR)				16/375 (4.3%)	
Tumor screening performed				114/375 (30.4%)	
Positive tumor screening among screened cases				18/114 (15.8%)	
History of status epilepticus				50/375 (13.3%)	
(**B**)					
**Autoimmune Thyroid Disease**	**T1DM Present**	**T1DM Absent**	**T1DM Not Reported**		**Total**
Present	23 (6.1%)	25 (6.7%)	14 (3.7%)		62 (16.5%)
Absent	16 (4.3%)	66 (17.6%)	3 (0.8%)		85 (22.7%)
Not reported	36 (9.6%)	47 (12.5%)	145 (38.7%)		228 (60.8%)
Total	75 (20.0%)	138 (36.8%)	162 (43.2%)		375 (100%)

Note: (**A**) Unilateral temporal lobe epilepsy was conservatively defined as an explicitly left- or right-temporal focus in the structured focal-lobe field. Extra-temporal MRI involvement was assigned only when explicit non-temporal neuroanatomic structures were described. Additional antibody positivity and the top three antibody frequencies were based on structured non-GAD antibody testing results; antibody testing was not systematic across all cases. (**B**) Percentages are based on all 375 cases.

**Table 3:** Immunotherapy exposure, assignable initiating regimen class, and timing data availability.

Characteristics	Value (%)
Cases with documented immunotherapy use	248/375 (66.1%)
Up-front regimen class assignable among treated cases	204/248 (82.3%)
Steroid plus IVIG up-front regimen	104/204 (51.0%)
Steroid-only up-front regimen	37/204 (18.1%)
IVIG-only up-front regimen	22/204 (10.8%)
Rituximab-based up-front regimen	16/204 (7.8%)
Maintenance-antimetabolite-based up-front regimen	15/204 (7.4%)
Steroid plus PLEX/immunoadsorption up-front regimen	7/204 (3.4%)
Apheresis-based up-front regimen	3/204 (1.5%)
Timing data available among treated cases	113/248 (45.6%)
≤6 months from symptom onset	70/113 (61.9%)
>6 to ≤12 months from symptom onset	4/113 (3.5%)
>12 months from symptom onset	39/113 (34.5%)
Timing data unavailable among treated cases	135/248 (54.4%)

Note: Initial immunotherapy regimens were grouped into mutually exclusive categories according to the treatment recorded at, or immediately around, immunotherapy initiation. Treatment timing was measured from symptom onset to the first documented immunotherapy. The intermediate >6 to ≤12 month group is shown for descriptive completeness but is excluded from the prespecified primary comparison of early (≤6 months) versus late (>12 months) treatment.

**Table 4:** Within-class early-versus-late immunotherapy timing analyses for the primary binary seizure outcome. Results are presented separately for the first post-immunotherapy assessment and for the last follow-up to improve readability.

(**A**) First evaluable post-immunotherapy seizure outcome				
**Treatment-Component Class (Within-Class Comparison; Rows Not Additive)**	**Early Immunotherapy (≤6 Months), Good Outcome**	**Late Immunotherapy (>12 Months), Good Outcome**	**Early Minus Late Difference (Percentage Points, 95% CI)**	***p*** **Value**
Steroid-containing	44/52 (84.6%)	15/17 (88.2%)	−3.6 (−18.4 to 20.1)	1.000
Apheresis/PLEX/immunoadsorption-containing	8/8 (100%)	3/7 (42.9%)	57.1 (11.5 to 84.2)	0.0256
Rituximab/CD20/B-cell-containing	15/16 (93.8%)	4/8 (50.0%)	43.8 (7.7 to 72.7)	0.0277
Cyclophosphamide-containing	2/2 (100%)	2/2 (100%)	0.0 (−65.8 to 65.8)	1.000
Escalation group (cyclophosphamide and/or CD20/B-cell/rituximab and/or tocilizumab)	17/18 (94.4%)	5/9 (55.6%)	38.9 (6.3 to 68.1)	0.0297
Azathioprine/mycophenolate/tacrolimus-containing	12/13 (92.3%)	2/6 (33.3%)	59.0 (14.2 to 83.5)	0.0173
Maintenance-after-episode regimens	14/17 (82.4%)	2/6 (33.3%)	49.0 (5.5 to 75.3)	0.0450
(**B**) Last available follow-up seizure outcome				
**Treatment-Component Class (Within-Class Comparison; Rows Not Additive)**	**Early Immunotherapy (≤6 Months), Good Outcome**	**Late Immunotherapy (>12 Months), Good Outcome**	**Early Minus Late Difference (Percentage Points, 95% CI)**	***p*** **Value**
Steroid-containing	44/53 (83.0%)	15/17 (88.2%)	−5.2 (−20.1 to 18.7)	1.000
Apheresis/PLEX/immunoadsorption-containing	7/8 (87.5%)	3/6 (50.0%)	37.5 (−9.1 to 70.4)	0.2448
Rituximab/CD20/B-cell-containing	15/16 (93.8%)	4/8 (50.0%)	43.8 (7.7 to 72.7)	0.0277
Cyclophosphamide-containing	2/2 (100%)	2/2 (100%)	0.0 (−65.8 to 65.8)	1.000
Escalation group (cyclophosphamide and/or CD20/B-cell/rituximab and/or tocilizumab)	17/18 (94.4%)	5/9 (55.6%)	38.9 (6.3 to 68.1)	0.0297
Azathioprine/mycophenolate/tacrolimus-containing	12/13 (92.3%)	3/6 (50.0%)	42.3 (1.9 to 74.2)	0.0709
Maintenance-after-episode regimens	15/18 (83.3%)	3/6 (50.0%)	33.3 (−5.2 to 66.4)	0.1391

Note: Early and late treatment were compared within each immunotherapy component class, using ≤6 months and >12 months from symptom onset as the predefined timing cutoffs. Component classes were allowed to overlap, because a single patient could receive a regimen containing multiple immunotherapy components; therefore, rows should not be summed. Effect estimates are the unadjusted absolute differences in good outcomes between early and late treatment, with 95% confidence intervals shown to indicate precision. Given the small number of late-treated cases in several classes, these findings should be regarded as exploratory and hypothesis-generating. The table is divided into two panels to improve readability.

**Table 5:** Epilepsy duration and selected baseline correlates of good seizure outcome at the first post-immunotherapy assessment and at the last follow-up.

(**A**) First evaluable post-immunotherapy seizure outcome				
**Predictor/Comparison**	**Group 1**	**Group 2**	**Effect Estimate**	***p*** **Value**
Epilepsy duration before immunotherapy	Patients with good outcome: 1.33 years (IQR 0.17–5.0)	Patients without good outcome: 5.0 years (IQR 1.0–14.25)	Not applicable	0.00086
Drug-resistant epilepsy	Drug-resistant cases: 54/82 with good outcome (65.9%)	Non-drug-resistant cases: 18/18 with good outcome (100.0%)	−34.1 percentage points (95% CI: −44.9 to −14.2)	0.0026
Tumor screening documented	Screening documented: 66/86 with good outcome (76.7%)	Screening not documented/performed: 54/91 with good outcome (59.3%)	+17.4 percentage points (95% CI: 3.6 to 30.2)	0.0159
(**B**) Last available follow-up seizure outcome				
**Predictor/Comparison**	**Group 1**	**Group 2**	**Effect Estimate**	***p*** **Value**
Epilepsy duration before immunotherapy	Patients with good outcome: 1.5 years (IQR 0.2–8.0)	Patients without good outcome: 4.0 years (IQR 1.0–9.0)	Not applicable	0.0241
Drug-resistant epilepsy	Drug-resistant cases: 65/89 with good outcome (73.0%)	Non-drug-resistant cases: 19/19 with good outcome (100.0%)	−27.0 percentage points (95% CI: −37.0 to −8.3)	0.0063
Tumor screening documented	Screening documented: 72/89 with good outcome (80.9%)	Screening not documented/performed: 60/93 with good outcome (64.5%)	+16.4 percentage points (95% CI: 3.4 to 28.6)	0.0196

Note: For epilepsy duration, which is a continuous variable, the table compares the median duration before immunotherapy between patients with good and poor seizure outcomes. For binary variables, the table compares the proportion of patients with good seizure outcomes among those with the given feature versus the corresponding comparison group. Effect estimates for binary variables are unadjusted absolute differences in good outcome rates, calculated as Group 1 minus Group 2; negative values indicate a lower rate of good outcomes in Group 1. Confidence intervals are exploratory and are provided to show the precision of the estimates. Tumor-screening status is included as an exploratory variable and should not be interpreted as a stable biological prognostic marker.

**Table 6:** Sodium-channel-blocking antiseizure medication exposure and seizure outcome after immunotherapy, stratified by immunotherapy timing.

**Timing Subgroup**	**Outcome Time Point**	**SCB-Exposed Cases with Good Outcome**	**Non-SCB-Exposed Cases with Good Outcome**	**Three-Category *p* Value**	**Binary *p* Value**
Early immunotherapy (≤6 months)	First post-immunotherapy assessment	17/18 (94.4%)	24/28 (85.7%)	0.612	0.634
Early immunotherapy (≤6 months)	Last follow-up	18/19 (94.7%)	23/28 (82.1%)	0.421	0.378
Late immunotherapy (>12 months)	First post-immunotherapy assessment	10/13 (76.9%)	7/11 (63.6%)	0.194	0.659
Late immunotherapy (>12 months)	Last follow-up	10/13 (76.9%)	8/10 (80.0%)	0.161	1.000

Note: Sodium-channel-blocking exposure was defined as treatment with lamotrigine, carbamazepine, oxcarbazepine, or valproate at or around immunotherapy initiation. Classification was based primarily on the antiseizure medication regimen recorded at immunotherapy initiation and secondarily on the structured antiseizure medication worksheet when direct treatment-start data were unavailable. Only 87 of 109 timing-evaluable treated cases could be classified for SCB exposure, and outcome-evaluable denominators were smaller in the subgroup analyses. The three-category *p* value compares seizure-free outcome, responder without seizure freedom, and non-responder outcome distributions between SCB-exposed and non-exposed cases. The binary *p* value compares good versus not good seizure outcomes. All analyses in this table are exploratory.

## Data Availability

Data are derived from a curated patient-level literature database. Included source publications are listed in Supplementary File S2 and additional source register audit details are provided in Supplementary File S1. Additional details about variable definitions and analytic decisions are available on request.

## Supplementary Materials

The following supporting information can be downloaded at: https://www.mdpi.com/article/10.3390/neurolint18060121/s1, Supplementary File S1 contain expanded descriptive tables (Tables S1–S10) for the literature-derived GAD65 antibody-associated epilepsy cohort; Supplementary File S2 lists the 132 source publications represented in the patient-level case database.
