# Supplementary material for: Timing, Composition, and Clinical Correlates of Immunotherapy Response in GAD65 Antibody-Associated Epilepsy: A Literature-Derived Patient-Level Analysis of 375 Published Cases
Source: Neurol Int. 2026 Jun 22;18(6):121. doi: 10.3390/neurolint18060121 (PMC13305307; doi:10.3390/neurolint18060121)
Supplement: Supplementary file 1 [file neurolint-18-00121-s001.zip › neurolint-4307755-Supplementary File S1.pdf]

Supplementary File S1: Expanded descriptive tables for the literature-derived GAD65 antibody-associated epilepsy cohort

Supplementary Table S1. Cohort provenance and demographic characteristics

Characteristic	Value
Unique cases	375
Source publications represented	132
Publication year window	1988-2026
Sex data available	347/375 (92.5%)
Female sex	253/347 (72.9%)
Male sex	94/347 (27.1%)
Adult-onset cases (>=18 years)	170/375 (45.3%)
Pediatric-onset cases (<18 years)	91/375 (24.3%)
Female sex among adult-onset cases	134/169 (79.3%)
Male sex among adult-onset cases	35/169 (20.7%)
Female sex among pediatric-onset cases	68/91 (74.7%)
Male sex among pediatric-onset cases	23/91 (25.3%)
Age at disease onset, years, mean (range)	25.0 (1.0-80.0)

Supplementary Table S2. Clinical, MRI, serologic, and oncologic characteristics

Characteristic	Value
Drug-resistance information available	210/375 (56.0%)
Drug-resistant epilepsy among informative cases	179/210 (85.2%)
Autoimmune thyroid disease only	25/375 (6.7%)
Type 1 diabetes mellitus only	16/375 (4.3%)
Both autoimmune thyroid disease and type 1 diabetes mellitus	23/375 (6.1%)
Definite temporal lobe epilepsy	223/375 (59.5%)
Unilateral temporal lobe epilepsy	99/375 (26.4%)
Bilateral temporal MRI involvement	63/375 (16.8%)
Extra-temporal MRI involvement	44/375 (11.7%)
Inflammatory MRI abnormalities	135/375 (36.0%)
Definite unilateral hippocampal sclerosis	46/375 (12.3%)
MRI-negative	99/375 (26.4%)
MRI data unavailable	62/375 (16.5%)
Multifocal epilepsy / multifocal involvement	65/375 (17.3%)
Very high GAD65 titer (serum and/or CSF)	224/375 (59.7%)
Intrathecal GAD65 positivity	87/375 (23.2%)
Any additional structured antibody positivity	75/375 (20.0%)
Most frequent additional structured antibody: Anti-TPO	34/375 (9.1%)
Second most frequent additional structured antibody: Anti-thyroglobulin	17/375 (4.5%)
Third most frequent additional structured antibody: AMPAR	16/375 (4.3%)
Tumor screening performed	114/375 (30.4%)
Positive tumor screening among screened cases	18/114 (15.8%)
History of status epilepticus	50/375 (13.3%)

Supplementary Table S3. Immunotherapy exposure and treatment-sequence classification

Characteristic	Value
Cases with documented immunotherapy use	248/375 (66.1%)
Steroid monotherapy only	32/248 (12.9%)
First-line-only immunotherapy	108/248 (43.5%)

Characteristic	Value
First- and second-line immunotherapy	96/248 (38.7%)
Second-line-only / other sequence	6/248 (2.4%)
Regimen-sequence unclassifiable from structured fields	38/248 (15.3%)
Timing informative among treated cases	113/248 (45.6%)
Early immunotherapy (<=12 months)	74/113 (65.5%)
Late immunotherapy (>12 months)	39/113 (34.5%)
Timing unavailable among treated cases	135/248 (54.4%)

Note. This table uses the <=12-month early-treatment descriptive framework retained from the original descriptive analyses.

Supplementary Table S4. Seizure outcomes according to immunotherapy timing (<=12 months vs >12 months)

Group	Cases, n	Early outcome evaluable, n	Non-responder	Responder	Seizure-free	Last follow-up evaluable, n	Non-responder	Responder	Seizure-free
Early IT (<=12 months)	74	60	10/60 (16.7%)	23/60 (38.3%)	27/60 (45.0%)	60	10/60 (16.7%)	22/60 (36.7%)	28/60 (46.7%)
Late IT (>12 months)	39	31	10/31 (32.3%)	13/31 (41.9%)	8/31 (25.8%)	30	8/30 (26.7%)	13/30 (43.3%)	9/30 (30.0%)

Note. Early post-immunotherapy outcome was taken from the first evaluable outcome recorded in the immunotherapy-lines sheet. Last follow-up outcome was taken from the latest available follow-up entry per case.

Supplementary Table S5. Seizure outcomes according to immunotherapy regimen-sequence group

Regimen-sequence group	Cases, n	Early outcome evaluable, n	Non-responder	Responder	Seizure-free	Last follow-up evaluable, n	Non-responder	Responder	Seizure-free
Steroid monotherapy only	32	19	4/19 (21.1%)	8/19 (42.1%)	7/19 (36.8%)	21	4/21 (19.0%)	10/21 (47.6%)	7/21 (33.3%)
Other first-line-only IT	76	51	19/51 (37.3%)	17/51 (33.3%)	15/51 (29.4%)	51	17/51 (33.3%)	17/51 (33.3%)	17/51 (33.3%)
First- and second-line IT	96	76	21/76 (27.6%)	34/76 (44.7%)	21/76 (27.6%)	77	18/77 (23.4%)	37/77 (48.1%)	22/77 (28.6%)

Note. These are mutually exclusive case-level groups based on all structured immunotherapy lines for a given case.

Supplementary Table S6. Surgical management and postoperative outcomes

Characteristic	Value
Unique operated cases	55/375 (14.7%)
Resection procedures	43

Characteristic	Value
Neuromodulation procedures	12
LITT procedures	1
Time from onset to first surgery, months, median (IQR)	96.0 (36.0-121.0) [n=17]
Documented early postoperative seizure outcome	18/55 (32.7%)
Non-responder	5/18 (27.8%)
Responder	11/18 (61.1%)
Seizure-free	2/18 (11.1%)
Documented last follow-up seizure outcome	46/55 (83.6%)
Non-responder	15/46 (32.6%)
Responder	20/46 (43.5%)
Seizure-free	11/46 (23.9%)
Pathology documented	14/55 (25.5%)
Inflammatory pathology among cases with pathology	9/14 (64.3%)
Hippocampal sclerosis pathology among cases with pathology	6/14 (42.9%)

Note. Procedure counts are per intervention and may therefore exceed the number of operated cases.

Supplementary Table S7. Musicogenic-trigger subgroup

Characteristic	Musicogenic trigger present	Musicogenic trigger absent
Subgroup size	16	92
Autoimmune thyroid disease present	7/16 (43.8%)	18/92 (19.6%)
Type 1 diabetes mellitus present	6/16 (37.5%)	19/92 (20.7%)
Evaluable early post-IT outcome	7/16 (43.8%)	47/92 (51.1%)
Good early IT response	2/7 (28.6%)	34/47 (72.3%)
Seizure-free after IT	0/7 (0.0%)	16/47 (34.0%)

Note. Restricted to cases with documented musicogenic-trigger status (present or explicitly absent).

Supplementary Table S8. Descriptive characteristics of early versus late immunotherapy (<=12 months vs >12 months)

Variable	Early IT (<=12 months)	Late IT (>12 months)
Patients, n	74	39
Time from symptom onset to first IT, median (IQR), months	0.5 (0.08-2.0)	72.0 (42.0-120.0)
Range, months	0-12	15-396
First regimen: steroid + IVIG, n (%)	22 (29.7)	3 (7.7)
First regimen: steroid, n (%)	15 (20.3)	6 (15.4)
First regimen: IVIG, n (%)	4 (5.4)	5 (12.8)
First regimen: IVIG + maintenance antimetabolite, n (%)	0 (0.0)	4 (10.3)
Other first regimens, n (%)	33 (44.6)	21 (53.8)
Early post-IT outcome evaluable, n	56	31
Last follow-up outcome evaluable, n	57	30
Last follow-up duration available, n	61	23
Last follow-up duration, median (IQR), months	8.0 (3.0-18.0)	12.0 (5.0-19.0)
Range of last follow-up, months	0.23-108.0	2.0-108.0

Note. Percentages are column percentages. Regimen categories were based on the earliest immunotherapy line per case.

Supplementary Table S9. Early post-immunotherapy seizure outcomes (<=12 months vs >12 months)

Outcome	Early IT	Late IT	Unadjusted RR (95% CI)
Seizure freedom after IT	27/56 (48.2%)	8/31 (25.8%)	1.87 (0.97-3.60)
>=50% seizure reduction after IT	49/56 (87.5%)	22/31 (71.0%)	1.23 (0.96-1.58)

Note. Early post-immunotherapy outcomes were derived from the earliest recorded immunotherapy line per case. Risk ratios are unadjusted.

Supplementary Table S10. Seizure outcomes at last follow-up (<=12 months vs >12 months)

Outcome	Early IT	Late IT	Unadjusted RR (95% CI)
Seizure freedom at last follow-up	28/57 (49.1%)	9/30 (30.0%)	1.64 (0.89-3.00)
>=50% seizure reduction at last follow-up	49/57 (86.0%)	22/30 (73.3%)	1.17 (0.92-1.49)

Note. Last follow-up outcomes were derived from the last available follow-up record per case. Risk ratios are unadjusted.
