# Supplementary material for: Timing, Composition, and Clinical Correlates of Immunotherapy Response in GAD65 Antibody-Associated Epilepsy: A Literature-Derived Patient-Level Analysis of 375 Published Cases
Source: Neurol Int. 2026 Jun 22;18(6):121. doi: 10.3390/neurolint18060121 (PMC13305307; doi:10.3390/neurolint18060121)
Supplement: Supplementary file 1 [file neurolint-18-00121-s001.zip › neurolint-4307755-Supplementary File S2.pdf]

Supplementary File S2

This table lists the 132 source publications represented in the patient-level case database. Records are ordered alphabetically by first author.

No.	First author	Year	Study title	Journal	Identifier(s)
1	Abbas	2024	Unusual clinical manifestation of Anti-GAD65 encephalitis in a patient with a background of epilepsy presented with seizure and altered mental status: A case report and literature review	Pharmacy Practice	DOI: 10.18549/PharmPract.2024.4.3053
2	Abu-Abaa	2023	Anti-Glutamic Acid Decarboxylase (GAD) 65 Encephalitis Mistaken for Herpes Encephalitis and Hashimoto's Encephalitis (HE): A Case Report	Cureus	DOI: 10.7759/cureus.35365
3	Achour	2018	Severe dysautonomia as a main feature of anti-GAD encephalitis: Report of a paediatric case and literature review	European Journal of Paediatric Neurology	DOI: 10.1016/j.ejpn.2018.01.004; PMID: 29370977
4	Akaishi	2015	抗 glutamic acid decarboxylase 抗体に関連した側頭葉てんかん 4 例の臨床的特徴 [Clinical features of four cases of temporal lobe epilepsy associated with anti-glutamic acid decarboxylase antibody]	Clinical Neurology (Rinsho Shinkeigaku)	DOI: 10.5692/clinicalneurol.cn-000740
5	Akin	2017	Autoimmune Limbic Encephalitis Associated with Type 1 Diabetes Mellitus	Journal of Clinical Research in Pediatric Endocrinology	DOI: 10.4274/jcrpe.3818
6	Akman	2009	Limbic encephalitis associated with anti-GAD antibody and common variable immune deficiency	Developmental Medicine & Child Neurology	DOI: 10.1111/j.1469-8749.2008.03217.x
7	Al-Attas	2021	Musicogenic reflex seizure with positive antiglutamic decarboxylase antibody: A case report	Epilepsia Open	DOI: 10.1002/epi4.12518
8	AlKhaja	2018	Conjoint glutamic acid decarboxylase 65 and P/Q voltage gated calcium channel antibodies in autoimmune epilepsy: A case report	Epilepsy & Behavior Case Reports	DOI: 10.1016/j.ebcr.2017.12.002
9	Alghamdi	2024	Co-existence of anti-glutamic acid decarboxylase-65 and anti-sry-like high-mobility group box receptor antibody-associated autoimmune encephalitis: A rare case report	Epilepsy & Behavior Reports	DOI: 10.1016/j.ebr.2024.100648
10	Aung	2025	Nine-Year Follow-Up of GAD65 Antibody Limbic Encephalitis With Clinical Remission Despite Persistent Imaging and Serological Abnormalities	Cureus	DOI: 10.7759/cureus.85726
11	Bach	2023	Co-occurrence of Anti-GAD65 Syndrome, Type 1 Diabetes Mellitus, and Focal Seizures With Impaired Awareness	Cureus	DOI: 10.7759/cureus.40611
12	Blanc	2009	Acute limbic encephalitis and glutamic acid decarboxylase antibodies: A reality?	Journal of the Neurological Sciences	DOI: 10.1016/j.jns.2009.09.004
13	Boesen	2019	Pediatric autoimmune encephalitis in Denmark during 2011-17: A nationwide multicenter population-based cohort study	European Journal of Paediatric Neurology	DOI: 10.1016/j.ejpn.2019.03.007; PMID: 31128894
14	Boronat	2011	GABAB receptor antibodies in limbic encephalitis and anti-GAD-associated neurologic disorders	Neurology	DOI: 10.1212/WNL.0b013e31820e7b8d
15	Brunker	2020	New-Onset Refractory Status Epilepticus with Underlying Autoimmune Etiology: a Case Report	SN Comprehensive Clinical Medicine	DOI: 10.1007/s42399-019-00185-z
16	Bushati	2022	Recurrent anti-GAD65 limbic encephalitis in a pediatric patient	Medico Research Chronicles	DOI: 10.26838/MEDRECH.2022.9.3.602
17	Carreño	2017	Epilepsy surgery in drug resistant temporal lobe epilepsy associated with neuronal antibodies	Epilepsy Research	DOI: 10.1016/j.eplepsyres.2016.12.010
18	Chen	2025	Anti-GAD65 Antibodies Related Refractory Epilepsy Successfully Treated with Tocilizumab: A Case Report and Systematic Literature Review	ImmunoTargets and Therapy	DOI: 10.2147/ITT.S520026
19	Chen	2025	Glutamic Acid Decarboxylase 65 Antibody-associated Epilepsy and Diplopia: Two Case Reports with Literature Review	The Cerebellum	DOI: 10.1007/s12311-024-01768-w
20	Chen	2025	Resolution of anti-GAD-associated autoimmune encephalitis in patients treated with efgartigimod	Frontiers in Neurology	DOI: 10.3389/fneur.2025.1550023
21	Chen	2026	Neuroinflammation in GAD65 Antibody-Associated Epilepsy Measured Using [18F]DPA-	Annals of Clinical and Translational	DOI: 10.1002/acn3.70324

			714 PET/MRI	Neurology	
22	Chengyu	2020	Clinical features and immunotherapy outcomes of anti-glutamic acid decarboxylase 65 antibody-associated neurological disorders	Journal of Neuroimmunology	DOI: 10.1016/j.jneuroim.2020.577289
23	Chou	2013	Limbic Encephalitis in Taiwanese Children and Adolescence: A Single Center Study	Pediatrics and Neonatology	DOI: 10.1016/j.pedneo.2013.01.016
24	Cianci	2010	Non-paraneoplastic limbic encephalitis characterized by mesio-temporal seizures and extratemporal lesions: A case report	Seizure	DOI: 10.1016/j.seizure.2010.06.002
25	D'Souza	2018	GAD65 antibody-associated autoimmune epilepsy with unique independent bitemporal-onset ictal asystole	Epileptic Disorders	DOI: 10.1684/epd.2018.0971
26	Di Giacomo	2019	Predictive value of high titer of GAD65 antibodies in a case of limbic encephalitis	Journal of Neuroimmunology	DOI: 10.1016/j.jneuroim.2019.577063
27	Di Giacomo	2025	Anti-GAD65 musicogenic epilepsy: Bilateral and independent mesial temporal seizures revealed by foramen ovale electrodes	Epilepsia Open	DOI: 10.1002/epi4.13132
28	Dimova	2022	Case Report: Multisystem Autoimmune and Overlapping GAD65-Antibody-Associated Neurological Disorders With Beneficial Effect of Epilepsy Surgery and Rituximab Treatment	Frontiers in Neurology	DOI: 10.3389/fneur.2021.756668
29	Douma	2021	Autoimmune Encephalitis in Tunisia: Report of a Pediatric Cohort	Journal of Immunology Research	DOI: 10.1155/2021/6666117; PMID: 34056010
30	Dubey	2014	Effectiveness of multimodality treatment for autoimmune limbic epilepsy	Epileptic Disorders	DOI: 10.1684/epd.2014.0703
31	Elisak	2018	The prevalence of neural antibodies in temporal lobe epilepsy and the clinical characteristics of seropositive patients	Seizure	DOI: 10.1016/j.seizure.2018.09.009
32	Errichiello	2009	Autoantibodies to glutamic acid decarboxylase (GAD) in focal and generalized epilepsy: A study on 233 patients	Journal of Neuroimmunology	DOI: 10.1016/j.jneuroim.2009.04.010
33	Falip	2012	Prevalence and immunological spectrum of temporal lobe epilepsy with glutamic acid decarboxylase antibodies	European Journal of Neurology	DOI: 10.1111/j.1468-1331.2011.03609.x
34	Falip	2017	Musicogenic reflex seizures in epilepsy with glutamic acid decarbocylase antibodies	Acta Neurologica Scandinavica	DOI: 10.1111/ane.12799
35	Fallatah	2025	GAD65-positive autoimmune-associated epilepsy presenting with ictal hand kissing: an uncommon presentation of a rare disease	Epilepsy & Behavior Reports	DOI: 10.1016/j.ebr.2025.100801; PMID: 40688008
36	Fan	2022	Clinical Heterogeneity in Acute Symptomatic Seizures due to Autoimmune Encephalitis Related to GAD65 Antibodies	Neuroimmunomodulation	DOI: 10.1159/000519229
37	Farooqi	2015	Therapeutic plasma exchange and immunosuppressive therapy in a patient with anti-GAD antibody-related epilepsy: quantification of the antibody response	Journal of Clinical Apheresis	DOI: 10.1002/jca.21342; PMID: 24961613
38	Fattahzadeh Ardalani	2024	Successfully treated anti-GAD limbic encephalitis in a 15-year-old diabetic boy with intravenous immunoglobulin: case report	Annals of Medicine & Surgery	DOI: 10.1097/MS9.0000000000001653
39	Fauser	2015	Long latency between GAD-antibody detection and development of limbic encephalitis – a case report	BMC Neurology	DOI: 10.1186/s12883-015-0435-9
40	Feyissa	2020	Brain-responsive neurostimulation treatment in patients with GAD65 antibody–associated autoimmune mesial temporal lobe epilepsy	Epilepsia Open	DOI: 10.1002/epi4.12395
41	Finelli	2011	Autoimmune Limbic Encephalitis With GAD Antibodies	The Neurohospitalist	DOI: 10.1177/1941875211413135
42	Gagnon	2016	Refractory status epilepticus and autoimmune encephalitis with GABAAR and GAD65 antibodies: A case report	Seizure	DOI: 10.1016/j.seizure.2016.02.006
43	Gardner	2016	GAD-65 Limbic Encephalitis Presenting as New-Onset Refractory Seizures	Journal of Clinical Medicine Research	DOI: 10.14740/jocmr2624w
44	Georgieva	2014	Cerebellar ataxia and epilepsy with anti-GAD antibodies: treatment with IVIG and plasmapheresis	BMJ Case Reports	DOI: 10.1136/bcr-2013-202314
45	Gillinder	2018	Refractory epilepsy secondary to anti-GAD encephalitis treated with DBS post SEEG evaluation: a novel case report based on stimulation findings	Epileptic Disorders	DOI: 10.1684/epd.2018.0993
46	Giometto	1998	Temporal-lobe epilepsy associated with glutamic-acid-decarboxylase autoantibodies	Lancet	DOI: 10.1016/S0140-6736(05)79192-3;

					PMID: 9708763
47	Grilo	2016	Type 1 diabetes and GAD65 limbic encephalitis: a case report of a 10-year-old girl	Journal of Pediatric Endocrinology and Metabolism	DOI: 10.1515/jpem-2016-0016
48	Hass	2026	Duolingo-induced seizures in GAD65 IgG associated autoimmune epilepsy	Epilepsy & Behavior Reports	DOI: 10.1016/j.ebr.2026.100848
49	Heiry	2015	Improvement of GAD65-associated autoimmune epilepsy with testosterone replacement therapy	Neurology: Neuroimmunology & Neuroinflammation	DOI: 10.1212/NXI.0000000000000142
50	Incecik	2018	Autoimmune encephalitis associated with glutamic acid decarboxylase antibodies: a case series	Acta Neurologica Belgica	DOI: 10.1007/s13760-018-0880-5
51	Jaafar	2020	Super refractory status epilepticus secondary to anti-GAD antibody encephalitis successfully treated with aggressive immunotherapy	Epilepsy & Behavior Reports	DOI: 10.1016/j.ebr.2020.100396
52	Janszky	2024	Management of autoimmune temporal lobe epilepsy with GAD65 antibody: four case reports	Neurologia i Neurochirurgia Polska	DOI: 10.5603/pjnns.98738
53	Jesus-Ribeiro	2020	Autoimmune musicogenic epilepsy associated with anti-glutamic acid decarboxylase antibodies and Stiff-person syndrome	Clinical Case Reports	DOI: 10.1002/ccr3.2538
54	Juneja	2022	An Evidence-Based Approach to Diagnosis and Prognosis in a Young Woman with New-Onset Super-Refractory Status Epilepticus: A Case Report	Case Reports in Neurology	DOI: 10.1159/000519947
55	Kanter	2008	Cyclophosphamide for anti-GAD antibody-positive refractory status epilepticus	Epilepsia	DOI: 10.1111/j.1528-1167.2007.01500.x
56	Kauppila	2019	Anti–Glutamic Acid Decarboxylase Encephalitis Presenting With Choreo-Dystonic Movements and Coexisting Electrographic Seizures	Movement Disorders Clinical Practice	DOI: 10.1002/mdc3.12800
57	Kern	2021	Rare presentation of anti-GAD-65 antibody-positive autoimmune encephalitis and simultaneous onset of type 1 diabetes mellitus in a paediatric patient	BMJ Case Reports	DOI: 10.1136/bcr-2020-237913; PMID: 33737275
58	Ketabi	2026	Anti-gad autoimmune encephalitis: a case report	BMC Neurology	DOI: 10.1186/s12883-026-04715-w
59	Khawaja	2016	Refractory status epilepticus and glutamic acid decarboxylase antibodies in adults: presentation, treatment and outcomes	Epileptic Disorders	DOI: 10.1684/epd.2016.0797
60	Kobayakawa	2010	A case of immune-mediated encephalopathy showing refractory epilepsy and extensive brain MRI lesions associated with anti-glutamic acid decarboxylase antibody	Clinical Neurology (Rinsho Shinkeigaku)	DOI: 10.5692/clinicalneurol.50.92; PMID: 20196490
61	Kojima	2014	PET-positive extralimbic presentation of anti-glutamic acid decarboxylase antibody-associated encephalitis	Epileptic Disorders	DOI: 10.1684/epd.2014.0666
62	Kopczak	2017	GAD antibody-associated limbic encephalitis in a young woman with APECED	Endocrinology, Diabetes & Metabolism Case Reports	DOI: 10.1530/EDM-17-0010
63	Korff	2011	Encephalitis Associated With Glutamic Acid Decarboxylase Autoantibodies in a Child: A Treatable Condition?	Archives of Neurology	DOI: 10.1001/archneurol.2011.177; PMID: 21825244
64	Kumar	2013	Histopathological evidence that hippocampal atrophy following status epilepticus is a result of neuronal necrosis	Journal of the Neurological Sciences	DOI: 10.1016/j.jns.2013.08.016
65	Kwan	2000	Glutamic acid decarboxylase autoantibodies in controlled and uncontrolled epilepsy: a pilot study	Epilepsy Research	DOI: 10.1016/S0920-1211(00)00180-7
66	Li	2020	Immune-mediated epilepsy with GAD65 antibodies	Journal of Neuroimmunology	DOI: 10.1016/j.jneuroim.2020.577189
67	Li	2022	Case report: Anti-GAD65 antibody-associated autoimmune encephalitis following HPV vaccination	Frontiers in Neurology	DOI: 10.3389/fneur.2022.1017086
68	Li	2024	Autoimmune encephalitis with coexisting antibodies to GABABR, GAD65, SOX1 and Ma2	BMC Neurology	DOI: 10.1186/s12883-024-03938-z
69	Li	2025	NORSE secondary to anti-GAD65 antibody-positive encephalitis treated with novel adjunctive rapid titration VNS protocol	Epilepsia Open	DOI: 10.1002/epi4.13096
70	Licchetta	2014	Limbic encephalitis with anti-GAD antibodies and Thomsen myotonia: a casual or causal association?	Epileptic Disorders	DOI: 10.1684/epd.2014.0668; PMID: 25036107
71	Liimatainen	2010	Clinical significance of glutamic acid decarboxylase antibodies in patients with epilepsy	Epilepsia	DOI: 10.1111/j.1528-1167.2009.02325.x

72	Lilleker	2014	Glutamic acid decarboxylase (GAD) antibodies in epilepsy: Diagnostic yield and therapeutic implications	Seizure	DOI: 10.1016/j.seizure.2014.04.009
73	Lin	2012	Antiglutamic Acid Decarboxylase Antibodies in Children With Encephalitis and Status Epilepticus	Pediatric Neurology	DOI: 10.1016/j.pediatrneurol.2012.06.013
74	Liu	2018	A Survival Case of Super-refractory Status Epilepticus due to Glutamic Acid Decarboxylase Antibodies-associated Limbic Encephalitis	Cureus	DOI: 10.7759/cureus.3125
75	Lopez-Sublet	2012	Limbic encephalitis and type 1 diabetes with glutamic acid decarboxylase 65 (GAD65) autoimmunity: Improvement with high-dose intravenous immunoglobulin therapy	Diabetes & Metabolism	DOI: 10.1016/j.diabet.2012.02.005
76	Luque-Llano	2025	A dual manifestation of GAD-antibody spectrum disorder: a case of progressive encephalomyelitis with rigidity, myoclonus and autoimmune epilepsy with mesial temporal sclerosis	BMC Neurology	DOI: 10.1186/s12883-025-04559-w
77	M'zahem	2016	Limbic encephalitis associated with glutamic acid decarboxylase antibodies in a young adolescent	Revue Neurologique	DOI: 10.1016/j.neurol.2016.04.001
78	Madkhali	2022	Intractable Seizures and Limbic Encephalitis, Unaccounted Complications of Type 1 Diabetes Autoimmunity	Journal of the Endocrine Society	DOI: 10.1210/jendso/bvab188
79	Mahesan	2024	Uncommon Pediatric Immune-Mediated Epilepsy: Disease Course, Diagnosis, and Outcome – A Series of Three Cases	Annals of Indian Academy of Neurology	DOI: 10.4103/aian.aian_149_24
80	Malter	2015	Treatment of immune-mediated temporal lobe epilepsy with GAD antibodies	Seizure	DOI: 10.1016/j.seizure.2015.05.017
81	Marchiori	2001	Encephalitis associated with glutamic acid decarboxylase autoantibodies	Neurology	DOI: 10.1212/WNL.56.6.814
82	Markakis	2014	Immunotherapy-responsive limbic encephalitis with antibodies to glutamic acid decarboxylase	Journal of the Neurological Sciences	DOI: 10.1016/j.jns.2014.05.032
83	Marnane	2008	New-onset focal epilepsy with palatal tremor and glutamic acid decarboxylase antibodies responding to intravenous immunoglobulin	Journal of Neurology	DOI: 10.1007/s00415-008-0986-4
84	Matà	2008	Non-paraneoplastic limbic encephalitis associated with anti-glutamic acid decarboxylase antibodies	Journal of Neuroimmunology	DOI: 10.1016/j.jneuroim.2008.05.015
85	McKnight	2005	Serum antibodies in epilepsy and seizure-associated disorders	Neurology	DOI: 10.1212/01.WNL.0000187129.66353.13
86	Mishra	2014	Anti–Glutamic Acid Decarboxylase Antibody Associated Limbic Encephalitis in a Child: Expanding the Spectrum of Pediatric Inflammatory Brain Diseases	Journal of Child Neurology	DOI: 10.1177/0883073813500527
87	Mohammadi-Asl	2025	Autoimmune Encephalitis and Musicogenic Epilepsy: A Case of GAD65 Antibody-Associated Seizure	Clinical Case Reports	DOI: 10.1002/ccr3.70444
88	Monnerat	2013	Opercular myoclonic-anarthric status epilepticus due to glutamic acid decarboxylase antibody-associated encephalitis	Epileptic Disorders	DOI: 10.1684/epd.2013.0596
89	Muñoz-Lopetegi	2020	Neurologic syndromes related to anti-GAD65: Clinical and serologic response to treatment	Neurology: Neuroimmunology & Neuroinflammation	DOI: 10.1212/NXI.0000000000000696
90	Mäkelä	2018	Clinical Management of Epilepsy With Glutamic Acid Decarboxylase Antibody Positivity: The Interplay Between Immunotherapy and Anti-epileptic Drugs	Frontiers in Neurology	DOI: 10.3389/fneur.2018.00579
91	Nagai	2019	Severe anti-GAD antibody-associated encephalitis after stem cell transplantation	Brain and Development	DOI: 10.1016/j.braindev.2018.10.006; PMID: 30381136
92	Najjar	2011	Extralimbic autoimmune encephalitis associated with glutamic acid decarboxylase antibodies: An underdiagnosed entity?	Epilepsy & Behavior	DOI: 10.1016/j.yebeh.2011.03.038
93	Niehusmann	2015	Non-paraneoplastic limbic encephalitis and central nervous HHV-6B reactivation: Causality or coincidence?	Neuropathology	DOI: 10.1111/neup.12283
94	Olson	2002	Type 1 diabetes mellitus and epilepsia partialis continua in a 6-year-old boy with elevated anti-GAD65 antibodies	Pediatrics	DOI: 10.1542/peds.109.3.e50; PMID: 11875178
95	Paredes-Aragón	2020	Continuous Visual Focal Status Epilepticus as the Primary Presentation of NMDA-R and GAD65-R Autoimmune Epilepsy	Frontiers in Neurology	DOI: 10.3389/fneur.2020.598974

96	Peltola	2000	Autoantibodies to glutamic acid decarboxylase in patients with therapy-resistant epilepsy	Neurology	DOI: 10.1212/WNL.55.1.46
97	Pondrelli	2022	Pilomotor seizures in autoimmune limbic encephalitis: description of two GAD65 antibodies- related cases and literature review	Seizure	DOI: 10.1016/j.seizure.2022.03.025
98	Randell	2018	Tocilizumab in refractory autoimmune encephalitis: a series of pediatric cases	Pediatric Neurology	DOI: 10.1016/j.pediatrneurol.2018.07.016; PMID: 30177347
99	Ren	2021	Case Report: Autoimmune Encephalitis Associated With Anti-glutamic Acid Decarboxylase Antibodies: A Pediatric Case Series	Frontiers in Neurology	DOI: 10.3389/fneur.2021.641024
100	Rácz	2022	Histopathologic Characterization and Neurodegenerative Markers in Patients With Limbic Encephalitis Undergoing Epilepsy Surgery	Frontiers in Neurology	DOI: 10.3389/fneur.2022.859868
101	Saidha	2010	Treatment of anti-glutamic acid decarboxylase antibody-associated limbic encephalitis with mycophenolate mofetil	Journal of Neurology	DOI: 10.1007/s00415-010-5476-9
102	Saiz	2008	Spectrum of neurological syndromes associated with glutamic acid decarboxylase antibodies: diagnostic clues for this association	Brain	DOI: 10.1093/brain/awn183
103	Salman	2021	GAD-65-Antibody-Associated Focal Epilepsy and Segmental Myoclonus in a Child	Canadian Journal of Neurological Sciences	DOI: 10.1017/cjn.2021.21
104	Sapana	2023	A case report of anti-GAD65 antibody-positive autoimmune encephalitis in children associated with autoimmune polyendocrine syndrome type-II and literature review	Frontiers in Immunology	DOI: 10.3389/fimmu.2023.1274672
105	Serrano-Castro	2023	Cenobamate in Patients With Anti-GAD65 Autoimmune-Associated Epilepsy	Neurology: Neuroimmunology & Neuroinflammation	DOI: 10.1212/NXI.0000000000200151
106	Shaaban	2025	New-onset drug-resistant epilepsy in type 1 diabetes mellitus patient following COVID-19 vaccination: suspicious for autoimmune pathogenesis	BMC Neurology	DOI: 10.1186/s12883-025-04126-3
107	Sharma	2012	GAD65 Positive Autoimmune Limbic Encephalitis: A Case Report and Review of Literature	Journal of Clinical Medicine Research	DOI: 10.4021/jocmr1080w
108	Sivathanu	2022	Favorable response to classic ketogenic diet in a child with anti-GAD65 antibody mediated super refractory status epilepticus	Epilepsy & Behavior Reports	DOI: 10.1016/j.ebr.2022.100557; PMID: 35789965
109	Smith	2021	Musicogenic epilepsy: Expanding the spectrum of glutamic acid decarboxylase 65 neurological autoimmunity	Epilepsia	DOI: 10.1111/epi.16888
110	Solimena	1988	Autoantibodies to glutamic acid decarboxylase in a patient with stiff-man syndrome, epilepsy, and type I diabetes mellitus	The New England Journal of Medicine	DOI: 10.1056/NEJM198804213181602; PMID: 3281011
111	Stagg	2010	Autoantibodies to glutamic acid decarboxylase in patients with epilepsy are associated with low cortical GABA levels	Epilepsia	DOI: 10.1111/j.1528-1167.2010.02644.x
112	Steriade	2025	Stereo-EEG associated anti-GAD65 autoimmune encephalitis – A report of two cases	Epilepsy & Behavior Reports	DOI: 10.1016/j.ebr.2025.100835
113	Suleiman	2013	Autoimmune epilepsy in children: Case series and proposed guidelines for identification	Epilepsia	DOI: 10.1111/epi.12142
114	Summers	2024	A Case of Anti-GAD65 Autoimmune Musicogenic Epilepsy	Practical Neurology	No DOI/PMID located
115	Thomsen	2026	Drug-resistant glutamic acid decarboxylase 65–associated epilepsy: Pre-surgical evaluation and comparison to temporal lobe epilepsy of other etiology	Epilepsia	DOI: 10.1002/epi.70068
116	Tian	2026	Case Report: Recurrent anti-glutamic acid decarboxylase 65 antibody-associated encephalitis in a child	Frontiers in Immunology	DOI: 10.3389/fimmu.2026.1729994
117	Tizazu	2020	Low rate of glutamic acid decarboxylase 65 (GAD-65) antibodies in chronic epilepsy	Seizure	DOI: 10.1016/j.seizure.2020.05.008
118	Triplett	2018	Fulminant Anti-GAD antibody encephalitis presenting with status epilepticus requiring aggressive immunosuppression	Journal of Neuroimmunology	DOI: 10.1016/j.jneuroim.2018.06.013
119	Tröscher	2023	Temporal lobe epilepsy with GAD antibodies: neurons killed by T cells not by complement membrane attack complex	Brain	DOI: 10.1093/brain/awac404
120	Valinčiutė	2023	GAD65 Antibody-Associated Epilepsy	Medicina	DOI: 10.3390/medicina59061135
121	Van Ael	2015	Anti-GAD antibodies, a rare cause of limbic encephalitis: a case report	Acta Neurologica Belgica	DOI: 10.1007/s13760-015-0493-1
122	Vegda	2023	GAD-65-Associated Limbic Encephalitis – Early Diagnosis and Course of Disease,	Annals of Indian Academy of	DOI: 10.4103/aian.aian_644_23

			Treated with IV Methylprednisolone	Neurology	
123	Vulliemoz	2007	Epilepsy and cerebellar ataxia associated with anti-glutamic acid decarboxylase antibodies	J Neurol Neurosurg Psychiatry	DOI: 10.1136/jnnp.2006.089268
124	Wang	2022	Psychotic Symptoms as the Initial Presentation of a Long-Lasting Misdiagnosed Anti-GAD65 Autoimmune Encephalitis: An Emblematic Case and Literature Review	Frontiers in Psychiatry	DOI: 10.3389/fpsyt.2022.754938
125	Widman	2015	Treating a GAD65 antibody-associated limbic encephalitis with basiliximab: a case study	Frontiers in Neurology	DOI: 10.3389/fneur.2015.00167
126	Xia	2023	Paraneoplastic anti-GAD65 extralimbic encephalitis presented with epilepsy: A case report	Medicine	DOI: 10.1097/MD.0000000000034780
127	Yang	2021	Glutamic acid decarboxylase 65-positive autoimmune encephalitis presenting with gelastic seizure, responsive to steroid: A case report	World Journal of Clinical Cases	DOI: 10.12998/wjcc.v9.i19.5325
128	Yoshimoto	2005	Type 1 diabetes mellitus and drug-resistant epilepsy: presence of high titer of anti-glutamic acid decarboxylase autoantibodies in serum and cerebrospinal fluid	Internal Medicine	DOI: 10.2169/internalmedicine.44.1174; PMID: 16357456
129	Zhang	2020	Clinical characteristics and outcomes of autoimmune encephalitis patients associated with anti-glutamate decarboxylase antibody 65	Clinical Neurology and Neurosurgery	DOI: 10.1016/j.clineuro.2020.106082
130	Zhang	2023	A Case of Anti-GAD 65 Autoimmune Encephalitis Associated with Focal Segmental Stiff-Person Syndrome	Brain Sciences	DOI: 10.3390/brainsci13020369
131	Zhang	2024	Epilepsia Partialis Continua in an Adolescent With GAD65 Antibody-Associated Encephalitis	Neurology	DOI: 10.1212/WNL.0000000000209981
132	Zhao-Fleming	2024	Outcomes of surgical resection and vagus nerve stimulation in patients with medically refractory epilepsy and glutamic acid decarboxylase 65 antibody positivity	Epilepsia	DOI: 10.1111/epi.18086
